# Editorial: Head and neck squamous cell carcinoma: navigating the dawn of personalized medicine

**DOI:** 10.3389/fonc.2025.1684617

**Published:** 2025-09-24

**Authors:** Luis Abel Quiñones, Ye Guo, Fujun Han

**Affiliations:** ^1^ Laboratory of Chemical Carcinogenesis and Pharmacogenetics (CQF), Department of Basic and Clinical Oncology, Faculty of Medicine, University of Chile, Santiago, Chile; ^2^ Department of Pharmaceutical Sciences and Technology, Faculty of Chemical and Pharmaceutical Sciences, University of Chile, Santiago, Chile; ^3^ Latin American Network for Implementation and Validation of Clinical Pharmacogenomics Guidelines (RELIVAF), Santiago, Chile; ^4^ Center for Cancer Prevention and Control (CECAN), Santiago, Chile; ^5^ Department of Oncology, Shanghai East Hospital, School of Medicine, Tongji University, Shanghai, China; ^6^ Cancer Centre, First Hospital of Jilin University, Changchun, China

**Keywords:** biomarker, head and neck neoplasms, precision therapy, artificial intelligence, personalized cancer therapy

Head and neck cancers (HNC), including squamous cell carcinoma (HNSCC) and rare subtypes, constitute a highly heterogeneous group of malignancies characterized by complex molecular profiles and wide variability in clinical outcomes (1–3). Recent advances in precision medicine, biomarker discovery, computational modelling, and pharmacovigilance have enabled significant progress in early diagnosis, therapeutic stratification, and the development of individualized treatment approaches. This Research Topic compiles nine articles that span a diverse array of contributions, from molecular biology and machine learning to clinical pharmacogenetics and adverse drug reaction monitoring, all aimed at improving outcomes for patients with head and neck malignancies.

In the context of tumor immunity, Lin et al. identified the Integrin Subunit Alpha L (ITGAL) as a pan-cancer biomarker associated with magnesium-mediated CD8+ T cell activation and immune infiltration in HNSCC, suggesting its potential as both a prognostic indicator and immunotherapy target. In parallel, Wang et al. reported that high cGAS-STING pathway activation enhances the efficacy of neoadjuvant chemo-immunotherapy in HNSCC, correlating with increased T cell infiltration and cytotoxic activity. Moreover, Zhou et al. characterized a four-gene signature linked to propionate metabolism in HNSCC, offering insights into immune evasion mechanisms and potential prognosis, and possible therapeutic targets.

Regarding therapy resistance, Chaudhary et al. identified *ACTL6A* and *ERCC1* as key chemoresistance genes in cisplatin-treated HNC, combining qPCR, bioinformatic modelling, and meta-analysis to propose drug repurposing strategies. Complementarily, He et al. demonstrated that aloe-emodin downregulates lncRNA D63785, thereby inhibiting the PI3K/Akt/mTOR axis in nasopharyngeal carcinoma, suggesting a novel pharmacological approach.

In the realm of prognostic modelling and treatment stratification, Liu et al. developed a ubiquitin-related gene signature for laryngeal cancer, linking it signatures to immune microenvironment modulation and treatment sensitivity. Similarly, Zhang et al. applied deep learning algorithms to personalize treatment in locally advanced HNSCC, enhancing survival prediction with performance comparable or superior to current clinical guidelines.

Addressing treatment safety, Gao et al. conducted an interesting pharmacovigilance study using WHO-VigiAccess (4), characterizing adverse drug reactions associated with five anti-HNSCC agents emphasizing the need for personalized safety monitoring.

Finally, Fan et al. reported a rare case of laryngeal sarcomatous carcinoma, reviewing molecular markers with potential therapeutic implications for aggressive HNC subtypes (5).

Together, these contributions highlight the multifaceted progress being made in head and neck oncology, with implications for biomarker-driven precision medicine, AI-supported clinical decision-making, and safety profiling. These advances are essential to improving both survival and quality of life for patients facing these challenging cancers.
